# Influence of the Fill Value Parameters on Acoustic and Physical–Mechanical Performance of 3D-Printed Panels

**DOI:** 10.3390/polym17131806

**Published:** 2025-06-28

**Authors:** Mihai Alin Pop, Mihaela Coșniță, Sebastian-Marian Zaharia, Lucia Antoaneta Chicoș, Cătălin Croitoru, Ionuț Claudiu Roată, Dorin Cătană

**Affiliations:** 1 Department of Materials Science, Transilvania University of Brasov, 500036 Brasov, Romania; mihai.pop@unitbv.ro; 2 Department of Product Design, Mechatronics and Environment, Transilvania University of Brasov, 29 Eroilor Ave., 500036 Brasov, Romania; 3 Department of Manufacturing Engineering, Transilvania University of Brasov, 500036 Brasov, Romania; zaharia_sebastian@unitbv.ro (S.-M.Z.); l.chicos@unitbv.ro (L.A.C.); 4 Materials Engineering and Welding Department, Transilvania University of Brasov, 500036 Brasov, Romania; c.croitoru@unitbv.ro (C.C.); ionut.roata@unitbv.ro (I.C.R.); catana.dorin@unitbv.ro (D.C.)

**Keywords:** 3D printing, infill density, polylactic acid (PLA), sound absorption, mechanical properties, acoustic insulation

## Abstract

This study investigates the acoustic and mechanical performance of three types of 3D-printed polylactic acid (PLA) panels with varying infill densities (5–100%) and structural configurations. Using fused filament fabrication (FFF), panels were designed as follows: Type 1 (core infill only), Type 2 (core infill + 1.6 mm shell), and Type 3 (core infill + multi-layer shells). Acoustic testing via impedance tube revealed that Type 2 panels with a 65% infill density achieved the highest sound absorption coefficient (α = 0.99), while Type 1 panels exhibited superior sound transmission loss (TLn = 53.3 dB at 60% infill). Mechanical testing demonstrated that shell layers improved tensile and bending resistance by 25.7% and 36.9%, respectively, but reduced compressive strength by 23.6%. Microscopic analysis highlighted ductile failure in Type 2 and brittle fracture in Type 3. The optimal panel thickness for acoustic performance was identified as 4 mm, balancing material efficiency and sound absorption. These findings underscore the potential of tailored infill parameters in sustainable noise-control applications.

## 1. Introduction

Noise pollution has emerged as one of the most pervasive environmental challenges of the 21st century, second only to air pollution in its detrimental impact on human health and ecosystems [1,2,3,4]. Chronic exposure to excessive noise levels—stemming from urbanization, industrial activities, and transportation—is linked to physiological ailments such as hypertension, sleep disturbances, and cognitive impairment, as well as psychosocial stressors that degrade quality of life [1]. While human auditory perception spans from 20 Hz to 20 kHz, low-frequency noise (20–500 Hz), often emitted by machinery and urban infrastructure, poses a particularly insidious threat due to its long wavelengths, which enable it to penetrate conventional sound barriers and persist over distances [5,6]. Mitigating these frequencies is critical for achieving acoustic comfort in buildings, vehicles, and public spaces, yet existing solutions remain inadequate.

Conventional porous absorbers, such as polyurethane (PU) foams and mineral wool, dominate the market due to their cost-effectiveness and ease of processing [7,8,9,10,11]. However, their efficacy diminishes at frequencies below 500 Hz, necessitating impractical thickness (often >10 cm) to achieve meaningful attenuation [7]. Furthermore, PU foams raise sustainability concerns: their petroleum-derived composition contributes to plastic waste accumulation, and their production involves toxic isocyanates, posing risks to both environmental and human health [8,12]. Alternative materials like glass foams or Portland cement, though effective in mid-frequency ranges, suffer from high carbon footprints and energy-intensive manufacturing processes [12]. These limitations underscore the urgent need for materials that reconcile acoustic performance, structural efficiency, and environmental sustainability.

In parallel, global regulatory frameworks, such as the EU’s Revised Energy Performance of Buildings Directive (2018) and the 2030 Noise Reduction Targets, now mandate stringent standards for energy efficiency and noise control in urban infrastructure [13,14]. These policies emphasize the development of lightweight, recyclable materials capable of attenuating low-frequency noise while minimizing embodied carbon. Biopolymers, particularly polylactic acid (PLA), have gained traction in this context. Derived from renewable resources like corn starch or sugarcane, PLA is biodegradable, recyclable, and compatible with additive manufacturing—a combination that aligns with circular economy principles [14,15,16]. While PLA’s mechanical properties and printability have been extensively studied for biomedical and packaging applications [17,18,19], its acoustic potential remains underexplored, especially in structured configurations optimized for noise control [20,21,22].

Additive manufacturing, particularly fused filament fabrication (FFF), offers unprecedented control over material architecture, enabling the design of porous geometries that dissipate sound energy through viscoelastic damping and Helmholtz resonance [23,24,25].

Recent papers have reported on 3D-printed lattice structures in which the influence of infill pattern on acoustic properties was investigated. The acrylonitrile butadiene styrene (ABS) prevails in these works due to its durability and versatility [26], and the pore shape variety refers to cubic, hexagonal, circular, triangular, rhomboid, orthogonal, starlite, etc., studied for their potential to broaden absorption bandwidths [5,27,28,29,30].

However, these designs often prioritize high-frequency performance, leaving low-frequency gaps, and neglect the interplay between infill density, shell reinforcements, and mechanical resilience [31,32,33,34]. For instance, enclosing infill structures with shells or varying layer thicknesses could enhance stiffness and redirect sound waves, but such configurations have not been systematically analyzed for PLA.

This study addresses critical gaps in our understanding of acoustic and mechanical performance in lightweight panel systems by systematically evaluating three innovative PLA panel architectures. The first design, referred to as Type 1, utilizes a core-only cubic infill structure that serves as a baseline model, providing a reference point for assessing open porosity and its effects. In contrast, the Type 2 design builds upon this foundation by incorporating a core infill that is encapsulated within a 1.6 mm thick perimeter shell. This configuration is deliberate in its intent: it aims to balance the inherent rigidity of the material with the need for adequate wave penetration, a factor essential for effective sound management. The most sophisticated of the three, Type 3, features a multi-layered design. It comprises five top and bottom shells, with each layer measuring 0.2 mm, integrated into a core–shell hybrid structure.

The objectives of this investigation are threefold. First, this study seeks to quantify the influence of varying infill densities—ranging from 5% to 100%—and the configuration of shell layers on key acoustic parameters, namely, sound absorption (α) and normalized transmission loss (TLn). This analysis places special emphasis on the low-frequency range of 500 to 1000 Hz, which is critical for many real-world noise reduction applications. Second, by correlating the observed acoustic performance with essential mechanical properties such as tensile, compressive, and flexural strengths, this research aims to identify and understand the trade-offs that arise between sound insulation and structural performance. Finally, this study endeavors to establish robust design guidelines for the fabrication of lightweight, sustainable PLA panels. These guidelines are intended not only to meet the stringent EU noise reduction targets but also to minimize material usage and environmental impact, ensuring both economic and ecological sustainability.

By integrating acoustic testing, mechanical characterization, and microscopic fracture analysis, this work advances the development of 3D-printed PLA as a multifunctional solution for noise-sensitive applications in architecture, automotive engineering, and public infrastructure.

## 2. Materials and Methods

### 2.1. Design of the Panels

The design of the specimens was carried out in the SolidWorks 2016 software system for acoustic testing, with the following dimensions: a diameter of 50 mm and thicknesses from 2 mm to 10 mm, dimensions that comply with both the standard and the technical characteristics of the impedance tube [35,36].

**Figure 1:**
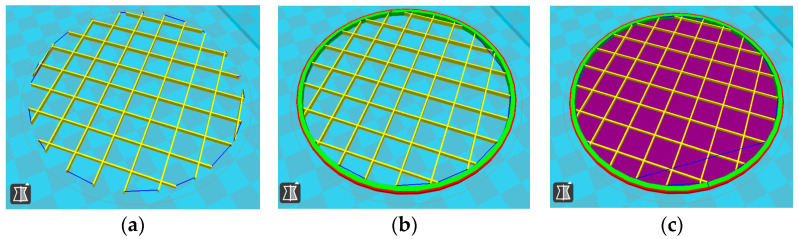
Parameters set for each type of specimen: (**a**)—Type 1 with 0 perimeters, 0 top layers, and 0 bottom layers; (**b**)—Type 2 with 4 perimeters, 0 top layers, and 0 bottom layers; (**c**)—Type 3 with 4 perimeters, 5 top layers, and 5 bottom layers.

**Figure 2:**
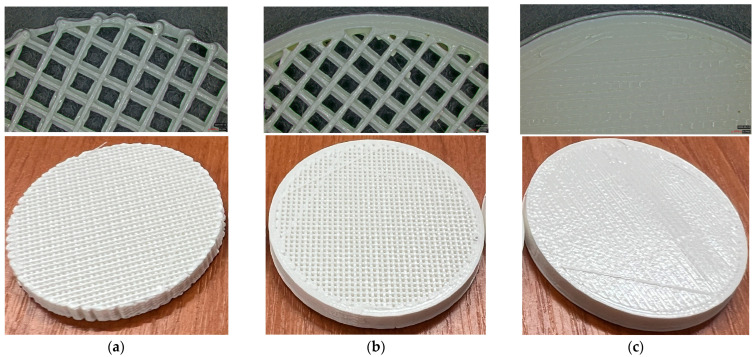
Types of 3D-printed panels: (**a**)—Type 1; (**b**)—Type 2; (**c**)—Type 3.

For each type of specimen, different sets of parameters (perimeter value and number of top and bottom layers) were established in the 3D printing slicing software CreatBot, V6.4.7, but at the same time, the other printing parameters remained constant (see Figure 1a–c and the 3D-printed panels presented in Figure 2).

### 2.2. Materials Properties

The specimens used for the experimental acoustic and mechanical tests were fabricated from standard white PLA [37,38]. Polylactic acid (PLA) is one of the most widely used thermoplastic polymers compatible with the material extrusion process. PLA offers several advantages [38,39,40]: it is a cost-effective and biodegradable material; it is highly suitable for FFF due to its ease of processing; it exhibits excellent dimensional stability, with minimal deformation during and after printing; it provides strong adhesion both to the build platform and between printed layers; and it does not emit unpleasant odors during processing. Furthermore, it demonstrates good first-layer adhesion during the printing process, enhancing print reliability [37,41,42].

### 2.3. Manufacturing Process of the 3D-Printed Panels

The 3D-printed panels were fabricated using the fused filament fabrication (FFF) technology with a CreatBot DX double-nozzle 3D printer (Henan Suwei Electronic Technology Co., Ltd., Zhengzhou, China). The printing parameters were selected based on the specific characteristics of the filament materials and were managed through the CreatBot slicing software, version V6.4.7. The establishment and choice of printing parameters were based on previous research conducted by the authors of this study [16,42,43], and the key 3D printing parameters employed in the fabrication of the panels are summarized in Table 1.

**Figure 3:**
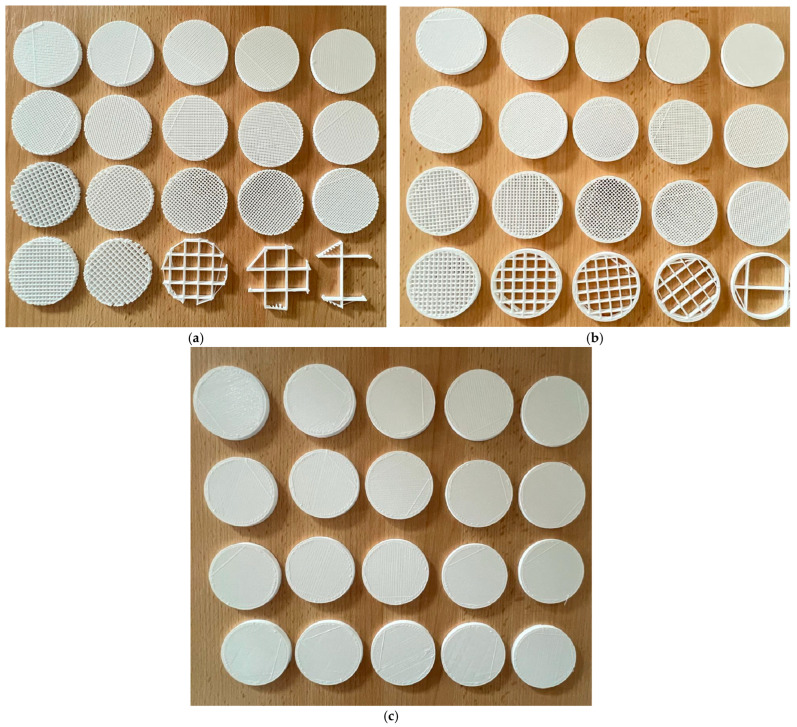
Three-dimensional-printed panels with all infill values: (**a**)—Type 1; (**b**)—Type 2; (**c**)—Type 3.

For each type of panel, the samples were printed with infill values of 5, 10, 15, …, and 100% and are presented in Figure 3.

**Figure 4:**
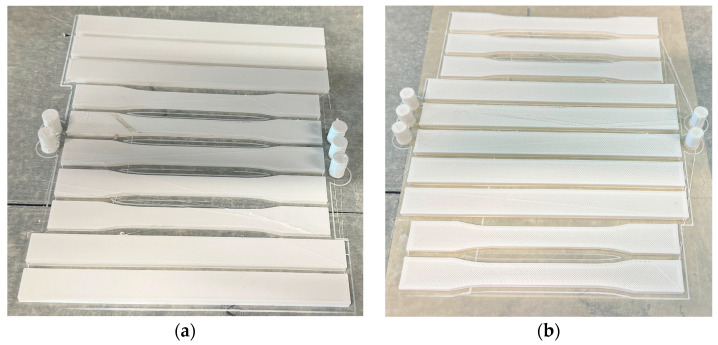
The 3D-printed samples used for mechanical testing on the build plate: (**a**) Type 3 with 60% infill and (**b**) Type 2 with 65% infill.

The sample design was carried out in the SolidWorks 2016 software system considering the standards specific to acoustic testing (ISO 10534-2 [36] and ASTM E1050 [37]) and for mechanical properties (Figure 4): ASTM D638-14 for tensile [42], ASTM D695-15 for compression [44], and ASTM D790-17 for three-point bending test [45].

The 3D-printed samples for acoustic testing have the following dimensions: the upper and lower parts have diameters of 50 mm and thicknesses between 2 and 10 mm.

### 2.4. Acoustic Testing

The behavior of samples fabricated using the fused filament fabrication (FFF) technology, which uses a material extrusion process to determine the sound absorption, was examined using a Holmarc HO-ED-A-03 acoustic impedance tube (Holmarc Opto-Mechatronics Ltd., Kochi, India). This setup comprises hollow tubes, two pairs of microphones, sample holders, a data acquisition system, and dedicated measurement software. The impedance tube itself is made of anodized aluminum with an internal diameter of 50 mm, allowing measurements within a frequency range of 500–3150 Hz.

**Figure 5:**
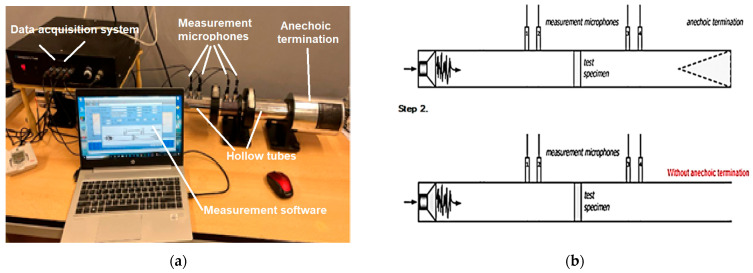
Experimental setup of the acoustic testing: (**a**) equipment used for acoustic testing of samples manufactured via the FFF technology; (**b**) method of measurement of the sound absorption coefficient and of the sound transmission loss [43].

In this study, the frequency-dependent characteristics of the sound absorption coefficient (α) and the sound transmission loss (STL) of the 3D-printed samples were analyzed using the transfer function method, in accordance with established standards [35,36]. Figure 5a illustrates the components of the impedance tube system employed for the acoustic measurements. Figure 5b presents the two schematic configurations of the system used to evaluate the acoustic properties of the samples: one setup includes an anechoic termination to measure the sound absorption coefficient, while the other excludes this termination to measure sound transmission loss. For each tested sample, relevant parameters—including sample geometry (50 mm diameter), microphone spacing (30 mm), as well as ambient temperature and humidity—were recorded during the measurements.

### 2.5. Mechanical Testing

Mechanical testing was conducted using a W-150 S universal testing machine (Jinan Testing Equipment IE Corporation, Jinan, China), equipped with a CELTRON PSD-20tSJTH force cell. For the mechanical tests, only the configurations with the best results (sound absorption and transmission loss) were chosen, namely, Type 2 with 65% infill and Type 3 with 60% infill.

For the experimental evaluation—flatwise compression and three-point bending—20 specimens were fabricated via the extrusion process, with five specimens per configuration (Type 2 and Type 3) for each test. This included 10 specimens for flatwise compression and 10 for three-point bending.

**Figure 6:**
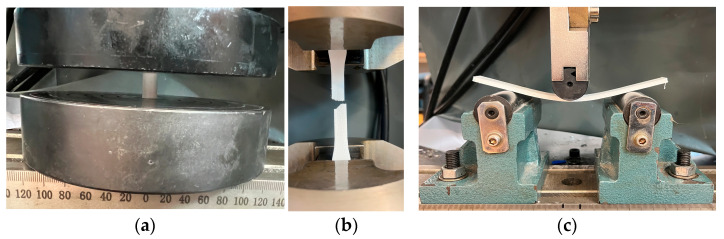
Mechanical testing: (**a**) compression testing; (**b**) tensile testing; (**c**) three-point bending testing.

Flatwise compression tests (Figure 6a) were performed in accordance with ASTM D695–15 [44] at a loading speed of 5 mm/min to assess the compressive strength and elastic modulus of the 3D-printed samples.

Three-point bending tests (Figure 6c) were conducted following ASTM D790–17 [45] at a crosshead speed of 5 mm/min to determine the flexural properties, including bending strength and load–displacement behavior.

Tensile tests (Figure 6b) were carried out to evaluate tensile strength, with a tightening speed of 5 mm/min and a stress rate of 10 MPa/s. A total of 10 tensile specimens (5 per configuration: Type 2 and Type 3) were tested in accordance with ASTM D638–14 [42].

All mechanical tests adhered to relevant standards, ensuring consistency in environmental conditions such as temperature and humidity.

### 2.6. Microscopic Analysis

Images of the fractured composite surfaces were taken using an optical microscope, type Leica, Emspira 3 model, Arnhem—Nederland.

## 3. Results and Discussion

### 3.1. The Effect of Printing Parameters on the Acoustic Performance

**Figure 7:**
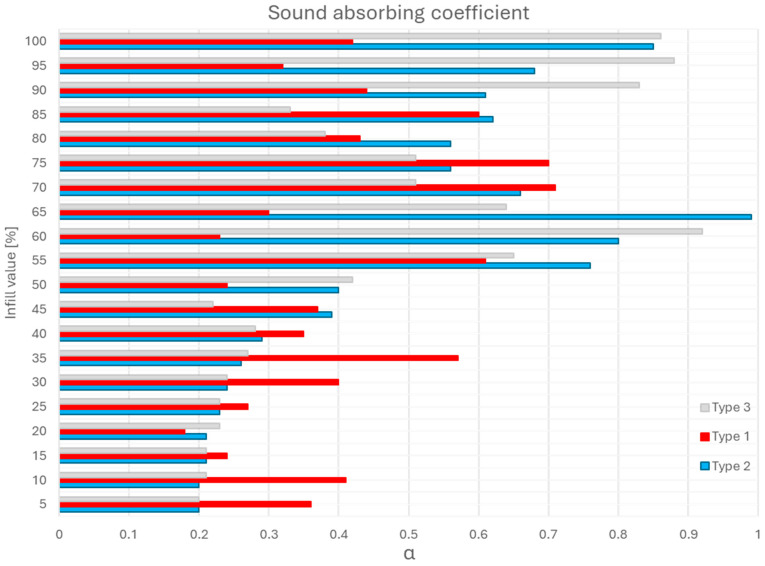
Sound absorbing coefficient function of infill value and sample type.

**Figure 8:**
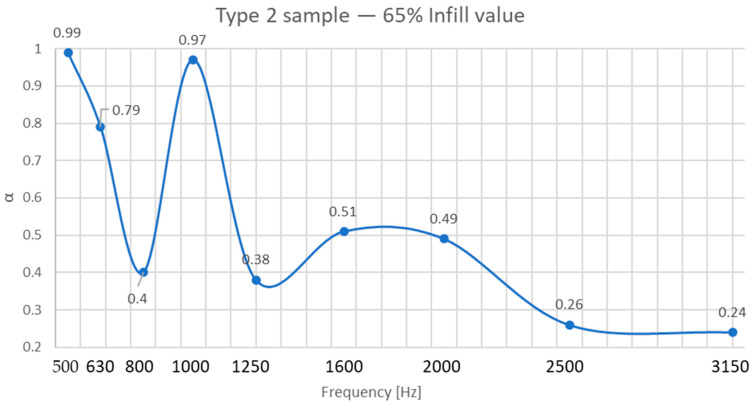
Sound-absorbing coefficient of Type 2 sample with 60% infill value.

As shown in Figure 7 and detailed in Figure 8, regarding the sound absorption coefficient depending on frequency, Type 2 panels (core infill + 1.6 mm shell) achieved the highest absorption coefficient (α = 0.99 at 65% infill), outperforming Type 1 (α = 0.92 at 60% infill) and Type 3 (α = 0.86 at 55% infill).

In the case of Type 1 panels, where we only have the structure given by the infill walls, the sound waves pass without encountering too much resistance at low filling densities but begin to be distributed throughout the sample mass as the filling level increases. This structure is less reliable and represents a design with lower acoustic absorption efficiency.

In the case of Type 2 panels, we obtained the best performance. This superiority stems from the interplay between open infill shape and the shell’s wave-redirecting effect. The cubic infill pattern at this density (65%) creates interconnected pores (≈0.5–1.2 mm diameter), enabling sound waves to penetrate and dissipate energy through viscoelastic damping and Helmholtz resonance, facts proven and presented by other authors in the specialized literature [27,31]. The 1.6 mm shell enhances structural rigidity, allowing partial wave reflection into the porous core.

With the increase in filling density, the parallelepipedal voids in the structure begin to merge, limiting the possibility of waves below 2000 Hz penetrating the structure, as other authors have shown in their research [7,12].

In the case of Type 3 panels, despite the additional top/bottom layers, excessive shell thickness (5 layers × 0.2 mm) restricted wave entry, reducing α by 7% compared to Type 2.

Notably, Type 2’s α = 0.99 surpasses conventional PLA panels (α = 0.6–0.85) [16] and rivals polyurethane foams (α = 0.95) [8], demonstrating the potential of hybrid core–shell designs for low-frequency applications.

This internal architecture at a density of 65% ensures high absorption at low to medium frequencies (500 Hz and 1000 Hz), being effective for absorbing noises such as voices or road traffic.

**Figure 9:**
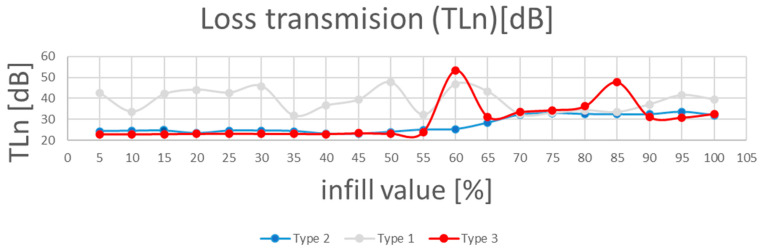
Transmission loss (TLn) coefficient function of infill value and sample Type.

Transmission loss behavior diverged from absorption coefficient trends (Figure 9). Type 3 panels exhibited the highest TLn (53.3 dB at 60% infill), outperforming Type 2 (TLn = 28.4 dB) and Type 1 (TLn = 47.6 dB).

**Figure 10:**
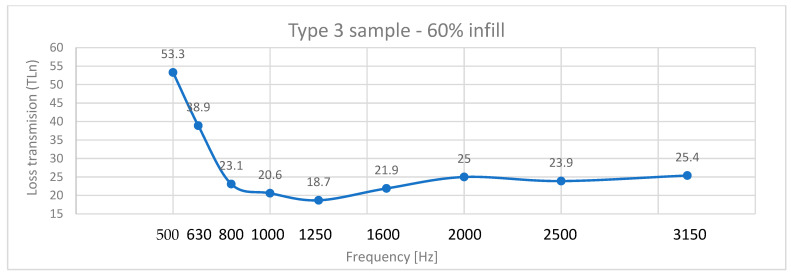
Transmission loss (TLn) coefficient of Type 3 sample—60% infill.

Type 3 panels, with five layers on the bottom and top as well as around the infill core, have the ability to cause greater sound loss due to the decrease in void size at the same time, with the growth of the frequency (Figure 10).

The Type 2’s perimeter shell introduces a TLn reduction, allowing partial transmission at shell–core interfaces. However, its TLn (28.4 dB) remains sufficient for applications requiring balanced absorption and insulation, such as HVAC duct liners.

Type 1 samples: the unshelled cores (60% infill) maximize material continuity, reflecting and scattering sound waves rather than absorbing them. The cubic lattice acts as a sound grating, attenuating specific frequencies through destructive interference [30].

Frequency dependence: TLn for all types increased linearly with frequency (R^2^ = 0.94), aligning with the mass law principle, where higher frequencies are easier to block [38].

**Figure 11:**
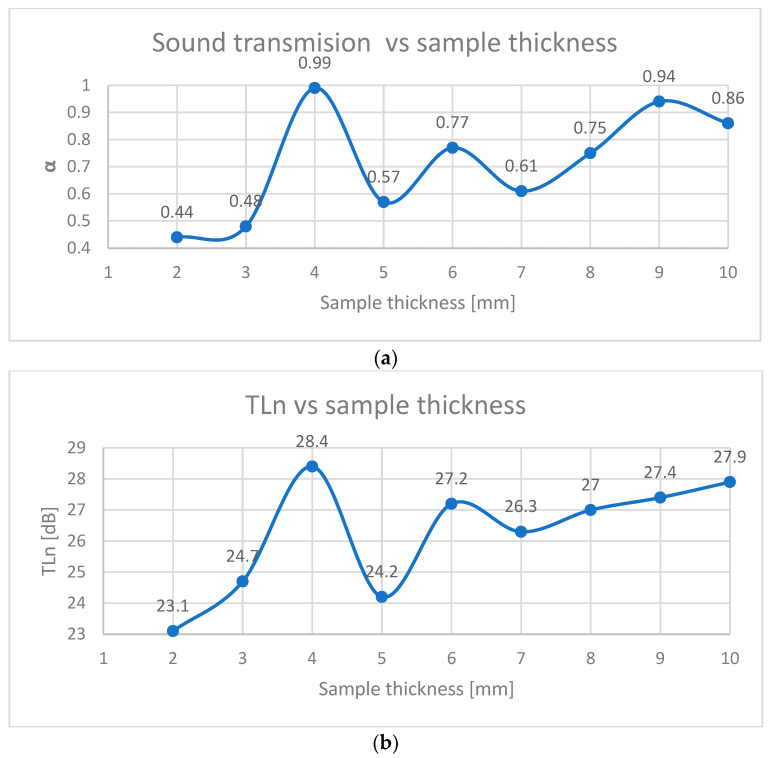
Sound absorbing properties versus thickness sample: (**a**) sound transmission vs. sample thickness; (**b**) TLn vs sample thickness.

To combine the material efficiency and performance, the thickness was optimized for Type 2 panels (65% infill), as illustrated in Figure 11a,b.

Peak α at 4 mm: A thickness of 4 mm achieved α = 0.99, as resonant frequencies of the panel aligned with the 500–1000 Hz range. Thinner panels (2–3 mm) lacked sufficient depth for wave dissipation, while thicker variants (6–10 mm) introduced excessive stiffness, reducing damping [7].

In the case of transmission loss, a maximum of 28.4 dB was reached in the case of 4 mm thick panels, after which there was a sudden drop in the case of 5 mm panels. Even though the values obtained subsequently started to increase with the thickness of the panels, the maximum of 28.4 dB was not reached.

These results indicate that the mass of the added material contributes very little to the sound-absorbing insulation.

Taking into account the consumption of material to obtain a panel with a surface of 1 m^2^ at a thickness of 4 mm (the best results obtained for TLn), in comparison with the consumption of material of the same surface but with a thickness of 10 mm (second place in terms of value of TLn), the reduced PLA consumption is 0.2 kg/m^2^, aligning with sustainability goals without compromising acoustic efficacy [3,14].

### 3.2. Physical–Mechanical Properties of 3D-Printed Panels

Mechanical tests demonstrated the following:

- Compression tests (Table 2) revealed a contrasting behavior, with shells reducing compressive resistance:

Type 2 with an average compressive strength (Rbc) = 36.6 MPa, 25.6% lower than Type 3 (49.2 MPa).

Microscopic analysis (Table 3) clarified this anomaly:

➢ The progressive compressing of the cubic infill structure absorbs the energy through plastic deformation. The ductility of the material facilitates this phenomenon, and due to end defects or interlayer pores, the deformation mechanism is unpredictable, resulting in slippage in the structure and variation in the obtained strength.
➢ The stress is localized at shell–infill interfaces, triggering premature buckling (Type 2) or interlayer delamination (Type 3) [35,37]. The rigid shell restricts infill deformation, limiting energy dissipation and accelerating collapse.

- Three-point bending tests highlighted the bonding between shells and infill under transverse loads:

Type 2 achieved a bending strength (R_bb_) of 78.6 MPa, which is 36.89% higher than Type 3 (49.6 MPa).

The multi-layered design of Type 2 optimally resists bending moments because the ttop/bottom shells have better resistance at compound tensile and compressive stresses, being the farthest from the neutral axis [2,27].

The core infill shape prevents shear failure by stabilizing shell layers, as evidenced by distributed microcracking in Type 2 vs. abrupt delamination in Type 3 [37,40].

Notably, Type 2’s maximum deflection (f) decreased by 13.72% (17.4 mm vs. Type 3’s 20.4 mm), underscoring the stiffening effect of shells at the expense of ductility.

- In the case of tensile tests, the following results were obtained:

Type 2 panels achieved an average tensile strength (R_m_) of 26.2 MPa, 25.19% higher than Type 3 (19.6 MPa).

This increase stems from two mechanisms:

- Stress redistribution: The perimeter shell (Type 2) and top/bottom layers (Type 3) mitigate stress concentrations at infill boundaries and delay crack initiation [25,40].
- Interlayer bonding: Shells act as continuous load-bearing paths, reducing reliance on infill parallelepiped structures in case of buckling under tension (Figure 3b) [16,38].

However, the deformation arrow (f) decreased by 13.72% in Type 2, indicating a stiffness–toughness trade-off typical of reinforced composites [19].

For all samples presented in Table 2, the standard deviations (ranging from 1.0 to 3.3) remain relatively negligible compared to their respective means. This is further confirmed by the coefficients of variation, which consistently fall below 10%. A coefficient of variation below this threshold signifies low variability in the experimental results, indicating that the data are well clustered around the mean and exhibit high homogeneity.

The mechanical–acoustic trade-offs are critical for application-specific optimization:

- Acoustic priority: Type 2 (65% infill + shell) balances a high α (0.99) with moderate tensile/compression strength, ideal for non-load-bearing soundproofing panels.
- Structural priority: Type 3 (60% infill) maximizes Rbc (78.6 Mpa) but sacrifices acoustic performance, suited for industrial flooring or machinery mounts, hybrid applications, or for partitions in modular architecture with intermediate properties (TLn = 28.4 dB).

### 3.3. Microscopic Analysis of the Panels

**Figure 12:**
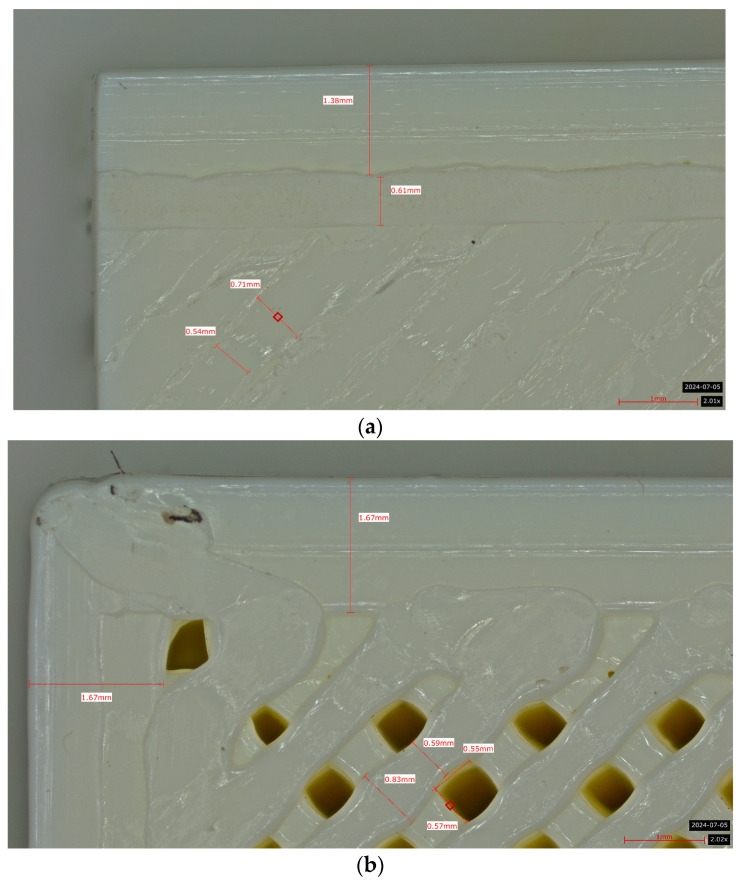
Microscopic analysis of the samples: (**a**) Type 3 sample and 60% infill; (**b**) Type 2 and 65% infill.

Microscopic evaluation of fractured surfaces (Figure 12, Table 3, Table 4 and Table 5) revealed distinct failure mechanisms across panel types, directly correlating with their structural configurations and mechanical performance. These findings provide critical insights into the interplay between infill architecture, shell reinforcement, and material behavior under stress.

As can be seen from Figure 12a, the surface does not present major defects but only a few and small end defects at the perimeter–infill interface.

In the case of Figure 12b, the same things are noted as in the case of Figure 12a, as well as the uniformity of the walls of the infill with the overlaps specific to the extrusion process (infill core).

Type 2 (65% Infill with Perimeter Shell):

In this case, the compressive failure is characterized by progressive buckling and localized crushing almost to the entire height of the structure (refer to Table 3—Type 2 sample, General view at 45° and general view from above). The cell walls exhibit a pronounced ductile deformation, plastic deformation, collapse, and folding, indicating significant energy absorption. Additionally, stress concentrations at the shell–core interfaces trigger minor delamination. However, this interaction maintains structural integrity until late-stage failure.

Type 3 (60% Infill with Multi-Layered Shells):

By contrast, the Type 3 configuration undergoes an abrupt brittle failure of approximately one-third of the base, with clean, planar cracks propagating through the infill nodes. This fracture mechanism is accompanied by minimal plastic deformation, due to the restricted mobility imposed by the multi-layered shell structure (refer to Table 3—Type 3 sample, General view at 45° and general view from above). Furthermore, weaker adhesion between layers, specifically delamination between the top and bottom shells and the core, acts as a stress concentrator, accelerating failure. This interlayer delamination is a crucial factor behind the 25.6% reduction in compressive strength compared to the Type 3 design (see Section 3.2), as shown in Table 3.

Type 2 panels: In Type 2 configurations, tensile failure is characterized by a mixed-mode fracture, with crack deflection occurring predominantly at the infill junctions (refer to Table 4, Type 2 sample—(a) fracture view from above).

A notable observation is the behavior of the material, like a composite with fiber, a phenomenon typical of brittle PLA.

The fracture at the edges of the specimen (in the shell) is almost perfectly straight, while inside the infill structure, it propagated in the areas with layer intersections.

Additionally, the continuous perimeter shell plays a crucial role in redistributing stress (refer to Table 4, Type 2 sample—(b) fracture view from above). By mitigating the pull-out of the infill walls, it enhances the overall tensile strength by 25.19% as detailed in Section 3.2, in comparison with Type 3 panels.

Type 3 panels: In contrast, Type 3 specimens exhibit a distinctly zig-zag brittle failure mode. The failure is dominated by interlayer delamination with minimal fibril formation, as shown in Table 4, Type 3 sample—(a) fracture view from above.

The weak adhesive bonding between the multi-layered structure and the core leads to clean, planar fractures (refer to Table 4, Type 3 sample—(b) fracture view from above), fractures that start/end in triangular end defects. This deficiency reduces the elongation at the break compared to Type 2, and the result is weaker. Moreover, microscopic analysis reveals the presence of voids approximately 50–100 µm in size located at the layer interfaces, PLA-specific triangular end defects, and adhesion defects between layers. These voids, and all defects in general, act as stress concentrators, further limiting the tensile performance.

Type 2 panels: Under three-point bending loads, Type 2 panels display a hybrid failure mode that combines the tensile and compressive failures, accompanied by the development of distributed microcracks nearly to the layer’s line intersection (refer to Table 5, Type 2 sample—(a) fracture view from above).

The integrity of the neutral axis is maintained as the perimeter shells effectively resist tensile stresses at the top and compressive stresses at the bottom, thereby mimicking the behavior of a composite beam (refer to Table 5, Type 2 sample—(b) fracture view from above). Additionally, the infill structure plays a reinforcing role by delaying shear-driven delamination, resulting in a 36.89% higher bending strength compared to the Type 3 configuration.

Type 3 panels: Conversely, Type 3 panels are prone to sudden delamination along the layers at the shell–core interface when subjected to bending. The rigidity of the multi-layer shells causes a stress concentration at the interfaces, intensifying the debonding process and breaking/cracking. The lack of ductile characteristics, such as fibril formation or crack branching, further underscores the brittle nature of the fracture (refer to Table 5, Type 3 sample—(a) fracture view from above and (b) fracture view from above). This behavior is reflected in an over 25% reduction in bending strength when compared to Type 2 panels.

#### Implications for Acoustic-Mechanical Synergy

The microscopic and mechanical analyses highlight two critical design insights:

- Shell Reinforcement Trade-offs: The incorporation of shells bolsters only strengths upon compression, improving them by 25.6%; this reinforcement introduces interfacial stress concentrations. These localized stresses diminish the tensile and three-point bending resistance and overall ductility.
- Infill Porosity Optimization: Open infill architectures, exemplified by Type 2 at 65% infill, promote energy dissipation through plastic deformation. This not only enhances acoustic damping capabilities but also contributes to increased mechanical toughness.

These findings are in alignment with previous studies on PLA composites [16,35] and underscore the necessity of balancing infill density and shell configuration in designing sustainable, high-performance acoustic panels.

## 4. Conclusions

The infill parameters, particularly infill density and pattern, play a significant role in determining the acoustic and mechanical properties of 3D-printed panels. These parameters are especially critical in applications such as soundproofing, structural components, and sustainable design.

In particular, the acoustic behavior of 3D-printed panels is strongly influenced by both infill design and panel thickness, as summarized in Table 6.

The parallelipedical arrangement of the infill pattern affects in an effective way the panel’s ability to absorb sound, creating resonant cavities that enhance absorption at specific frequencies.

In fields such as automotive and architectural design, where both mechanical strength and acoustic performance are required, optimizing infill parameters is essential. Choosing the appropriate infill pattern and density ensures that panels can withstand mechanical stresses while achieving the desired acoustic functionality.

The results of this study underscore the profound effect of infill parameters on the acoustic performance of PLA-based 3D-printed panels. The highest sound absorption coefficient (α = 0.99) was recorded for Type 2 panels printed with a 65% infill density and no top or bottom layers.

By contrast, panels printed with a 60% infill density and an enclosed infill architecture reached a slightly lower absorption value (α = 0.92), though still exceeding values commonly reported in the literature.

These findings highlight that both infill percentage and the presence or absence of surrounding layers critically influence acoustic performance. Open infill architectures with higher densities allow greater sound wave penetration and internal energy dissipation, resulting in enhanced sound absorption. Compared to traditional PLA acoustic panels, which typically exhibit α values ranging from 0.6 to 0.85, the tested configurations offer substantial improvements.

This study also found that a panel thickness of 4 mm is optimal, providing high sound absorption while reducing material consumption. For a 1 m^2^ panel area, this configuration uses 0.2 kg less material and achieves higher acoustic performance (α = 0.99) compared to similar panels with top and bottom layers and a perimeter (α = 0.92).

In the case of mechanical tests comparing Type 2 panels vs. Type 3 panels, the following results were obtained:

- At tensile, a 25.19% increase in resistance;
- At compression, a 25.6% decrease in resistance;
- At three-point bending, there is a 36.89% increase in resistance but a 13.72% decrease in bending deflection.

In conclusion, this study confirms that tuning infill parameters is a practical and effective strategy for designing customized, high-performance acoustic materials using additive manufacturing. This approach supports the development of sustainable and adaptive sound control solutions for architectural and industrial applications.

Acoustic–mechanical synergy plays a crucial role in this research by revealing the interdependent relationship between the acoustic performance and the physical–mechanical properties of the 3D-printed panels. Our study shows that optimizing for one property often involves trade-offs with another. For instance, while Type 2 panels with 65% infill exhibit excellent sound absorption, they offer moderate tensile strength (26.2 MPa) and compressive strength (36.6 MPa). This makes them ideal for non-load-bearing soundproofing applications where high absorption is the key parameter. Type 3 panels, designed for higher mechanical resistance (compressive strength of 49.2 MPa), showed a decrease in sound absorption. This highlights a direct trade-off: enhancing mechanical robustness by increasing material density or shell thickness can compromise acoustic absorption but can be suitable for hybrid applications or partitions in modular architecture.

Understanding this synergy allows for the design of multifunctional materials tailored to specific performance requirements. For example, selecting a Type 2 configuration would be suitable for room acoustics, while a Type 3 configuration could be more appropriate for industrial flooring or machinery mounts where structural integrity is critical, along with some noise reduction.

Our findings enable industries to design and manufacture panels specifically tailored for noise-sensitive applications across various sectors. In architecture and construction, these panels can be used for soundproofing walls, ceilings, and partitions in all types of buildings, as well as for creating acoustic diffusers and absorbers in spaces like auditoriums and recording studios. In automotive engineering, they support the development of lighter, more efficient sound-dampening components that enhance cabin comfort and fuel efficiency. For public infrastructure, our solutions are ideal for noise barriers along highways and railways or as acoustic treatments in transportation hubs. HVAC systems benefit from more efficient duct liners that curb noise transmission through ventilation. Importantly, our use of PLA—a biodegradable polymer aligns well with sustainable manufacturing strategies.

Building on our current findings, future research will focus on advancing the understanding and application of 3D-printed acoustic materials across several key areas. We aim to explore additional printing parameters—like nozzle diameter, speed, and layer height—to fine-tune design guidelines by examining their effects on acoustic and mechanical performance. To ensure real-world viability, we will assess the long-term durability, aging characteristics, and environmental resistance of PLA panels under diverse conditions. Moreover, we plan to examine scalable production techniques and perform detailed cost–benefit analyses to support industrial deployment.

## Figures and Tables

**Table 1:** Summary of the 3D printing parameters used in the fabrication of test panels via the FFF technology.

Parameter	Value
	**PLA**
Filament diameter	2.85 [mm]
Layer height	0.2 [mm]
Infill density	5; 10; 15; 20; 25; 30; 35; 40; 45; 50; 55; 60; 65; 70; 75; 80; 85; 90; 95; 100 [%]
Print speed	40 [mm/s]
Travel speed	120 [mm/s]
Printing temperature	230 [°C]
Building plate temperature	60 [°C]
Infill pattern	cubic
Hotend	0.6 [mm]

**Table 2:** Type 2 and Type 3 3D-printed panels—physical mechanical properties.

Sample	Statistical Indicators	Compression R_bc_ [MPa]	Three-Point Bending		Tensile R_m_ [MPa]
			R_bb_ [MPa]	f [mm]	
Type 2—65% Infill density	Average	36.6	78.6	17.6	26.2
	Standard deviation	2.1	2.3	1	2.4
	Coefficient of variation	5.7	2.9	5.5	9.1
Type 3—60% Infill density	Average	49.2	49.6	20.4	19.6
	Standard deviation	1.6	3.3	1.1	1.8
	Coefficient of variation	3.3	6.6	5.6	9.3

Where R_bc_ = Compression resistance [Mpa]; R_bb_ [Mpa] = Three-point bending resistance [Mpa]; f [mm] = arrow (deformation) [mm]; R_m_ [Mpa] = Tensile strength [Mpa].

**Table 3:** Microscopic analysis failure mode for samples subjected to compression stress.

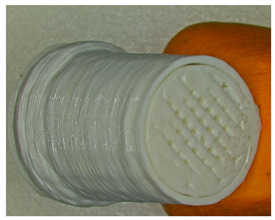 Type 2 sample—General view at 45°	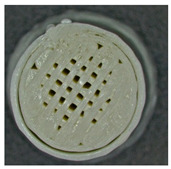
	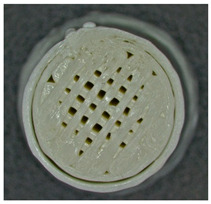 (a) General view from above 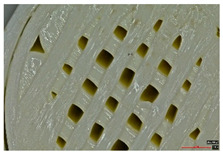 (b) Detailed top view
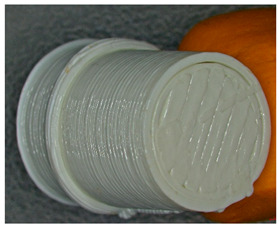 Type 3 sample—General view at 45°	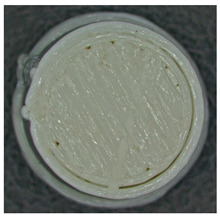 (a) General view from above
	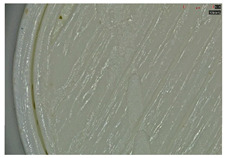 (b) Detailed top view

**Table 4:** Microscopic analysis failure mode for samples subjected to tensile stress.

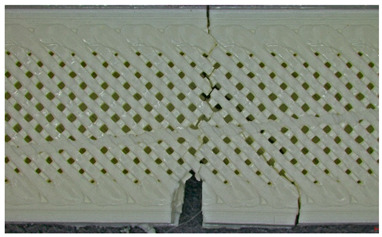 Type 2 sample—General view	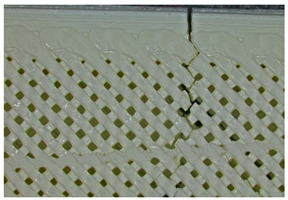 (a) Fracture view from above
	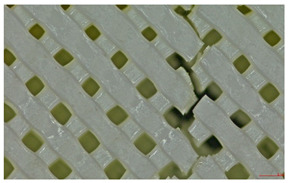 (b) Detailed fracture top view
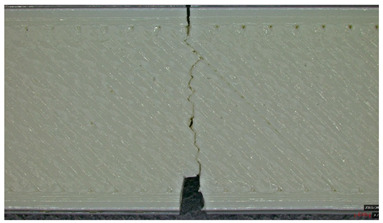 Type 3 sample—General view	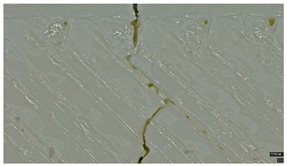 (a) Fracture view from above
	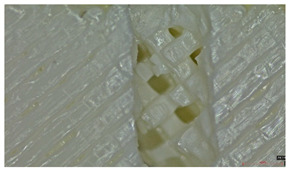 (b) Detailed fracture top view

**Table 5:** Microscopic analysis failure mode for samples subjected to three-point bending.

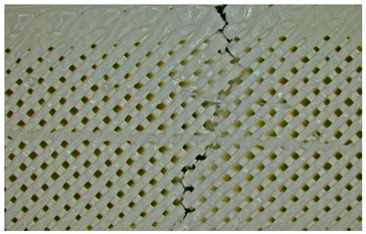 Type 2 sample—General view	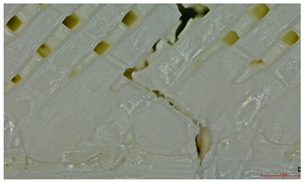 (a) Fracture view from above
	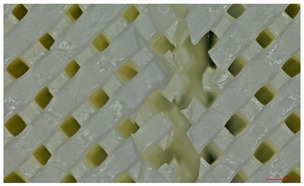 (b) Detailed fracture top view
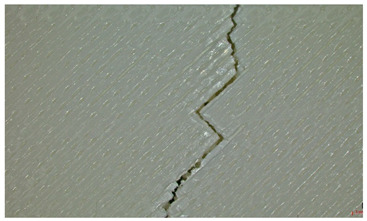 Type 3 sample—General view	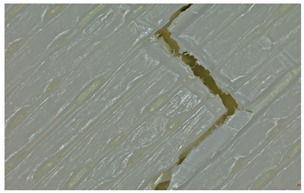 (a) Fracture view from above
	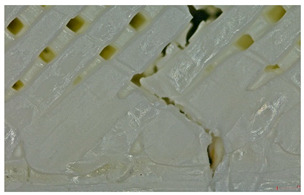 (b) Detailed fracture top view

**Table 6:** Summary of Acoustic Performance.

Property	Best Performing Infill (%)	Frequency Range	Notes
Sound Absorption (α)	Type 2—65%	800–1600 Hz	Best for mid-range frequencies
Transmission Loss (TLn)	Type 3—60%	2500–3150 Hz	Best for high-frequency insulation
Balanced Performance	65–75%	800–2500 Hz	Good absorption and moderate TLn

## Data Availability

Data are contained within the article.
